# Conserved QTL and chromosomal inversion affect resistance to columnaris disease in 2 rainbow trout (*Oncorhyncus mykiss*) populations

**DOI:** 10.1093/g3journal/jkac137

**Published:** 2022-06-06

**Authors:** Federico C F Calboli, Heikki Koskinen, Antti Nousianen, Clémence Fraslin, Ross D Houston, Antti Kause

**Affiliations:** Natural Resources Institute Finland (LUKE), FI-31600 Jokioinen, Finland; Natural Resources Institute Finland (LUKE), FI-70210 Kuopio, Finland; Natural Resources Institute Finland (LUKE), FI-70210 Kuopio, Finland; The Roslin Institute and Royal (Dick) School of Veterinary Studies, University of Edinburgh, Easter Bush EH25 9RG, UK; The Roslin Institute and Royal (Dick) School of Veterinary Studies, University of Edinburgh, Easter Bush EH25 9RG, UK; Natural Resources Institute Finland (LUKE), FI-31600 Jokioinen, Finland

**Keywords:** rainbow trout, columnaris disease, QTL, GWAS, inversion

## Abstract

We present a comparative genetic analysis of the quantitative trait loci underlying resistance to warm water columnaris disease in 2 farmed rainbow trout (*Oncorhynchus mykiss*) populations. We provide evidence for the conservation of a major quantitative trait loci on Omy03, and the putative role played by a chromosomal rearrangement on Omy05. A total of 3,962 individuals from the 2 populations experienced a natural *Flavobacterium columnare* outbreak. Data for 25,823 genome-wide SNPs were generated for both cases (fatalities) and controls (survivors). *F_ST_* and pairwise additive genetic relationships suggest that, despite being currently kept as separate broodstocks, the 2 populations are closely related. Association analyses identified a major quantitative trait loci on chromosome Omy03 and a second smaller quantitative trait loci on Omy05. Quantitative trait loci on Omy03 consistently explained 3–11% of genetic variation in both populations, whereas quantitative trait loci on Omy05 showed different degree of association across populations and sexes. The quantitative trait loci on Omy05 was found within a naturally occurring, 54.84 cM long inversion which is easy to tag due to a strong linkage disequilibrium between the 375 tagging SNPs. The ancestral haplotype on Omy05 was associated with decreased mortality. Genetic correlation between mortality in the 2 populations was estimated at 0.64, implying that the genetic basis of resistance is partly similar in the 2 populations. Our quantitative trait loci validation identifies markers that can be potentially used to complement breeding value evaluations to increase resistance against columnaris disease, and help to mitigate effects of climate change on aquaculture.

## Introduction

*Flavobacterium columnare* is a well-known infectious disease agent in freshwater fish farming (Newton *et al.* 1997; Waskiewicz and Irzykowska 2014). *Flavobacterium* *columnare* is the etiological cause of columnaris disease, which predominantly manifests itself as ulcerations of the gills and of the skin. Mortality due to columnaris disease is especially high in younger fish, where the disease produces an acute response, and depending on the virulence of the strain, untreated fry die between 12 and 48 h after infection (Declercq, Haesebrouck, *et al.* 2013, Evenhuis *et al.* 2014). The disease affects multiple freshwater fish species at warm water temperatures, typically above 20°C (Declercq, Haesebrouck, *et al.* 2013; Declercq, Boyen, *et al.* 2013), though it has been observed affecting salmonid species even at colder temperatures of 12–14°C (Starliper 2011). Climate change is increasing temperatures worldwide (Zachos *et al.* 2001), affecting inland water temperatures (van Vliet *et al.* 2013), with direct repercussions on water temperatures in fry ponds and tanks, potentially leading to more frequent columnaris outbreaks (Karvonen *et al.* 2010). Currently a live attenuated vaccine is being developed, but early testing on channel catfish (*Ictalurus punctatus*) suggests negligible effects on mortality (Malecki *et al.* 2021). The vaccine is not commercially available for salmonids. Disease management therefore relies on antibiotics (Laanto *et al.* 2015), the routine use and need of which is less than ideal, due to environmental impact concerns, and due to the risk of selection of antibiotic resistance (Declercq, Haesebrouck, *et al.* 2013; Laanto *et al.* 2015; Zhang *et al.* 2017). There is therefore a strong interest in selecting for improved resistance in breeding programs of aquaculture species, an approach that may adapt farmed fish also to climate change (Sae-Lim *et al.* 2017).

The ability to use high density, genome-wide marker panels for the aquaculture species of interest (either by using commercially available SNP chips, or genotyping by sequencing), allows assessment of the association between genomic regions and phenotype using a genome-wide association study (GWAS). Markers showing significant association with the phenotype are potentially linked to a quantitative trait locus (QTL) that is part of the genetic architecture of the trait. Causal variants of QTLs are not normally directly identified, and can be challenging to map without sufficiently dense marker sets. Additionally, larger structural polymorphisms such as chromosome inversions may create long chromosome regions in which markers are very tightly linked with limited recombination. Such regions may harbor QTLs for multiple traits and the limited recombination makes these regions “supergenes” in which long haplotypes with phenotypic effects on multiple traits exist (Villoutreix *et al.* 2021). It has been hypothesized that major QTLs may be more prominent in farmed fish compared to terrestrial livestock, perhaps due to the diversity of life-history strategies and the early phase of domestication in aquatic species (Houston *et al.* 2020).

There are several approaches to use QTL information in selective breeding. In genomic selection, genomic estimated breeding values (GEBV) are calculated using genome-wide information provided by tens of thousands of markers at once (Meuwissen *et al.* 2001; Goddard and Hayes 2009). In the genomic evaluation, phenotype–marker associations can be further emphasized by weighting the SNPs or genomic regions by their allele substitution effects on a phenotype (Wang *et al.* 2014) or by using Bayesian methods that model non-normal distribution of the SNP effects (Kärkkäinen and Sillanpää 2012). Alternatively, in marker-assisted selection (MAS), a few specific markers linked to major QTLs are used in the evaluation of individuals (Lande and Thompson 1990; You *et al.* 2020), and MAS can be easily combined with breeding value evaluations that are based on relationships among individuals (i.e. based on pedigree or realized genomic relationship matrix [GRM]).

Approaches using QTL information for genomic evaluation and selection are likely to be especially effective for those phenotypes that are oligogenic in origin, or where QTLs are within a chromosomal rearrangement and where recombination is suppressed, conditions that allow the use of few highly informative tagging markers. Moreover, specific QTL information is more useful in smaller data sets, and when marker density is low and does not to cover all linkage disequilibrium blocks across the genome (Misztal *et al.* 2020). To effectively use QTL information in a breeding program, it would be useful that the link between the markers and the phenotypic effect would be persistent. In farmed fish, only a few QTLs have been validated by replication in multiple studies so far (e.g. Houston *et al.* 2010; Barson *et al.* 2015; Gonen *et al.* 2015; Liu *et al.* 2018; Boison *et al.* 2019; Hillestad and Moghadam 2019; Aslam *et al.* 2020; Hillestad *et al.* 2020), and the understanding of the role of chromosomal inversions on determining trait variation is limited.

An important aquaculture species susceptible to columnaris disease is the rainbow trout (*Oncorhynchus mykiss*). Rainbow trout is a prominent aquaculture species worldwide due to its fast growth and adaptability to different environments (Boudry *et al.* 2021). This species has been the subject of extensive genetic analysis, which has generated a reference genome (Berthelot *et al.* 2014; Gao *et al.* 2018), a commercial SNP chip (Palti *et al.* 2015), and detailed information about naturally occurring genomic rearrangements, such as inversions (Pearse *et al.* 2019; Weinstein *et al.* 2019; Liu *et al.* 2021), that are present in this species. It is known that rainbow trout exhibits genetic-based differences in mortality caused by the bacterial cold-water disease (Leeds *et al.* 2010; Evenhuis *et al.* 2015; Liu *et al.* 2018), a disease caused by *Flavobacterium psychrophilum*, which is very closely related to *F.* *columnare*, and it has also been observed that rainbow trout exhibits a positive correlation between mortality due to the 2 diseases (Evenhuis *et al.* 2015; Silva, Evenhuis, Vallejo, Gao, *et al.* 2019; Silva, Evenhuis, Vallejo, Tsuruta, *et al.* 2019). This observation suggests that this species could harbor genetic variation and genetic variants that decrease mortality to columnaris disease infection. If that were the case, it would be valuable to understand the genetic basis of decreased mortality, so that this information can be incorporated in breeding programs (Kause *et al.* 2005).

Here we present the results of a genome-wide association analysis for mortality after an outbreak of *F. columnare* infection in rainbow trout from 2 Finnish broodstocks. The use of fish from 2 populations allowed us to specifically address the questions of (1) whether we would observe conservation across populations of any effects of chromosomal regions and inversions on mortality, (2) what the heritability of mortality to infection is in the 2 populations, and (3) whether we would observe a positive genetic correlation between mortality in the 2 populations. We used genomic information to calculate the realized marker-based genomic relationships between all samples, both within and between populations. This allowed us to obtain a direct measure of the genetic correlation for mortality between the 2 populations. We quantify the degree of genetic differentiation between the populations and discuss our results in the light of replicability of association analysis results of QTLs, and other genomic structural variants.

## Materials and methods

### Populations and family structure

The fish used in this study come from 2 broodstocks, one from Savon Taimen Oy, a Finnish rainbow trout breeder and fingerling producer based in Rautalampi, and the second from the national breeding program maintained by the Natural Resources Institute Finland (LUKE), based in Enonkoski research farm. The 2 broodstocks are located at separate farms but their offspring were reared on the same farm. Matings were performed and families established in May 2019 for both broodstocks. For the Savon Taimen samples, 100 dams and 25 sires (mating 4 dam to each sire) were used to establish 100 families in a farm located in East-Finland on the 2nd and 3rd of May. The Savon Taimen sires were sex reversed females, thus all the offspring were females. For the LUKE samples, 33 dams and 48 sires were used to establish 105 families (fertilizing the eggs from each dam with multiple sires) at the Enonkoski farm on the 15th of May. LUKE sires were natural males, and thus the offspring contained both sexes. A fin clip was collected from all sires and dams for genotyping.

For both populations separately, 0.5 dl of eggs was sampled from each mating and all families within a population were thereafter pooled. The eggs were sent to the farm of Hanka-Taimen Oy (Hankasalmi, Finland), and the eggs of the 2 populations were separately incubated and fingerlings reared in bulk. Each broodstock produced about 35,000 (Savon Taimen) and 31,000 (LUKE) fry, which were randomly distributed into 3 tanks for each population, 6 tanks in total. Density was hence an average of 100 fry from each family in each tank. All tanks were adjacent to one another, were subject to the same light conditions, and shared the same source of water from a nearby river.

The study started on June 24, 2019 when fry were moved into fingerling tanks. Across the whole study, all tanks were monitored daily for mortality and signs of any disease, and dead individual were collected once or twice a day. On July 4 and July 5, 2019, the first fish with signs of columnaris disease were observed. Only the fry showing clear signs of columnaris disease were randomly sampled among the fish that died in the first 5 days of the outbreak, and tail tissue was extracted and used for DNA analysis. Some of the samples were sent for veterinary examination, and once the presence of columnaris disease was confirmed, treatment was initiated to stop the outbreak on July 26. For both populations, 510 dead or dying fish were sampled for tissue from each tank. Fish rearing continued in the tanks after the end of the outbreak. On July 19, the fish were moved to new larger tanks, all fish from 1 tank to a 1 new tank.

In September–October 2019, all the remaining fish were counted and 510 surviving fish were sampled randomly from each tank.

The population-wide mortality to columnaris disease was 30% for the Savon Taimen, 10,650 dead fish over 34,951 fish in total, and 39% for the LUKE population, 12,476 dead fish over 31,696 fish in total. The overall mortality across the 2 populations was 35%.

### Genotyping

Of the samples collected as mentioned above, a total 1,450 offspring samples and 104 parents (1 parent was collected but accidentally not sent for genotyping, and the genotyping company genotyped 20 parents twice, omitting 20 unique parents) were genotyped for the Savon Taimen stock, and 3,055 offspring samples and 81 parents were genotyped for the LUKE stock.

For these samples DNA was extracted and genotyped by IdentiGen Ltd. using the commercially available 57K SNP Axiom Trout Genotyping Array (Palti *et al*. 2015). After genotyping, we used the Axiom Analysis Suite version 5.1.1 to call genotypes for all samples, and we exported the resulting genotypes as a PLINK format .ped and .map files for further analysis.

For all samples, the Axiom Array nominally provided data for 57,501 SNPs. After genotype calling, 36,227 SNPs were classified as being polymorphic at high resolution by the Axiom Suite and were retained. These SNPs were further filtered by removing all SNPs that mapped on unplaced scaffolds and SNPs that mapped to more than 1 position on the genome assembly, that had a missing rate of 5% or more, that had a *P*-value for Hardy–-Weinberg equilibrium smaller than 0.000001, and that had a Minor Allele Frequency of 0.05 or lesser. After these quality control steps our data comprised of 25,853 high-quality filtered SNPs for downstream analyses. We also removed all individual samples missing 10% or more of their genotypes, leaving 1,363 Savon Taimen samples (1,284 offspring and 79 parents), 2,772 LUKE samples (2,691 offspring and 81 parents), for a total of 4,135 samples after quality control. All markers were mapped against the Omyk_1.0 genome (Gao *et al.* 2018, accession number GCA_002163495.1), with each SNP position recorded on the Axiom database.

### Trait definition and sex phenotypes

Mortality was coded as a binary trait. Dead and dying offspring fish were coded as 1 and alive coded as 0. To determine the sex of LUKE offspring fish, we selected 7 sex-associated SNPs (Palti *et al.* 2015), and we used the LUKE parents, whose sex was known, to assess the sensitivity (true positive rate) and specificity (true negative rate) (Fawcett 2005) of these markers in identifying males. Once the specificity and sensitivity were assessed, we used the methods described in Toli *et al.* (2016), which uses a Bayesian approach, to determine whether a fish is male. Starting with a 0.5 prior, each fish was tested 1 SNP at a time to calculate the posterior probability that the fish is male, using the results of each test as the new prior to the following test. After 7 consecutive tests, we could assign a posterior probability of being male to each fish, with the posteriors being in all cases greater than 0.995 (for males), or smaller than 0.005 (for females). The final figures for the LUKE population were 1,341 males and 1,337 females. For the Savon Taimen fish, all offspring were female, so no specific sex determination was needed.

### Genomic relatedness of 2 populations

Using the SNP data, we quantified the genomic differentiation of the 2 populations using an *F_ST_* analysis, and the pairwise kinship between samples using the additive genetic relationship coefficient, excluding all parents in both analyses. *F_ST_* quantifies the partitioning of genetic diversity in the SNPs within and across populations, a value of zero implying panmixia and a value of 1 that all genetic variation is explained by the population structure. We used PLINK to calculate both measures (Chang *et al.* 2015). We used PLINK to provide the Weir and Cockeram *F_ST_* estimate (Weir and Cockerham 1984) by using the –fst option, and we calculated a full set of Identity-By-Descent measures including the pairwise additive genetic relationship (Weir *et al.* 2006) using the –genome option. The Weir pairwise additive genetic relationship is bound between 0 and 1 and can be directly be compared to pedigree-based relatedness analyses.

### Genome-wide association analyses

We used genome-wide complex trait analysis (GCTA) for genome-wide association analyses (Yang *et al.* 2011). Analyses were carried out on the offspring only because the parents of both populations did not experience the *F. columnare* outbreak and thus had no survival phenotype. We performed 2 kinds of genome-wide association analyses with mortality using GCTA. The first is a mixed linear model analysis in which GCTA calculates the GRM based on all SNPs, and then 1 SNP at a time and the GRM are simultaneously fitted in the model, together with covariates (-MLMA option). The random GRM factor controls for population structure and genomic relationships (Yang *et al.* 2011). The second is a mixed linear model with a leave 1 chromosome out (MLMA-LOCO) option. With this option, each candidate SNP is still fitted one at a time together with the covariates as described above, but rather than using the same GRM for each SNP, a new partial GRM is calculated every time by removing all the other SNPs in the same chromosome as the candidate SNP, and then calculating a new GRM on the fly (Yang *et al.* 2014). Yang *et al.* (2014) have shown this approach to increase the power of the analysis in those cases where the SNP under exam is not itself associated with the phenotype but linked to a SNP that is causal, because without this correction the causal SNP affects the analysis twice, once through linkage with the SNP in analysis, and once through the GRM, leading to a loss of power.

GWAS was performed within populations, and in all combinations of populations and sexes. The model used in the GWAS was:

y=a+bx+G+tank+sex+e (model 1)

with *y* the phenotype, *a* the mean value, *b* the fixed regression slope (beta) corresponding to the allele substitution effect of the SNP tested, *x* the SNP genotype (with levels 0/1/2 corresponding to the number of minor alleles in the genotype of an individual), *G* is the random genetic effect captured by the GRM, tank the fixed tank effect (with 3 levels within a stock, and 6 levels across the stocks), sex is the fixed effect for the sex of the fish (with 2 levels), and *e* is the random error. The GRM was calculated using either the MLMA or LOCO approach. When analyzing data on one sex only, the gender effect was excluded. Because the families were split across tanks, we did not investigate maternal effects.

The proportion of genetic variance explained by a SNP (%SNP $\sigma_{\text{SNP}}^{2}$) was calculated as (Lynch and Walsh 1997):

$$\%\sigma_{\text{SNP}}^{2}=p\left(1-p\right)b^{2}/\sigma_{\text{grm}}^{2}*\;100$$

in which *b* is as determined before, *p* is the frequency of the common allele, $\sigma_{\text{grm}}^{2}$ is the total genetic variance explained by the genomic relationships, estimated in GCTA with the null model:

y=a+G+tank+sex+e (model 2)

To correct for multiple statistical tests, we employed a genome-wide significance threshold of 1.9 × 10^−6^ (the Bonferroni corrected threshold of 0.05/25,823 SNPs), and a chromosome-wide significance threshold of 5.6 × 10^−5^ (the chromosome averaged Bonferroni correction 0.05/(25,823 SNPs/29 chromosomes)). These thresholds were applied to all analyses because in all analyses the number of SNPs were the same, and because the Bonferroni correction is dependent on the number of SNPs.

### Tagging SNPs for Omy5 inversion

We tested for the presence of a previously identified large inversion on chromosome Omy05 (Pearse *et al.* 2019), using tagging SNPs identified in the wild rainbow trout populations in the United States (Gao *et al.* 2021). Of the 475 SNPs listed by Gao *et al.* (2021) as fully tagging the inversion, 375 were common with our data, allowing us to identify unequivocally the genotype of each fish for the presence of the Ancestral (A) or Rearranged (R) haplotypes based on the identification of Pearse *et al.* (2019).

### Estimation of heritability and genetic correlation

To estimate the overall genetic variance in mortality within the populations and the genetic correlation between the populations, BLUPF90 was used (Misztal *et al.* 2002). Phenotypes of the 2 populations were coded to be 2 different traits. Thus, it was possible to calculate the genetic correlation between these 2 traits, and the *h*^2^ for mortality in each population.

For the estimation of the genetic parameters, we used 2 models. The first is the linear mixed model that estimates the heritability on the observed scale, as implemented in the AIREMLF90 module (which uses a maximum likelihood approach). Using the formula of Dempster and Lerner (1950), the observed, 0 and 1, scale estimate of heritability was corrected for the prevalence of mortality in each population to obtain the heritability at the underlying liability scale.

The second approach uses a threshold model that estimates *h*^2^ directly on an underlying normally distributed liability scale, as implemented by the THRGIBBS3F90 module (which uses Gibbs sampling). Posterior distribution of the parameters was obtained by Gibbs sampling using the THRGIBBS3F90 module run with a burn in of 250,000 iterations, a sampling run of 25,000 iterations that saved every 50th iteration.

A bivariate model was used in which the Model 2 was used for both population-specific traits, and the residual covariance between the traits was assumed to be zero. Heritability was computed as:

$$h_{i}^{2}=\sigma_{gi}^{2}/(\sigma_{gi}^{2}+\sigma_{e}^{2})$$

with $h_{i}^{2}$ the heritability in population *i*, $\sigma_{gi}^{2}$ the genetic variance for mortality for population *i*, and $\sigma_{e}^{2}$ the residual variance, and the genomic correlation was

$$cor_{ij}=cov_{ij}/\sqrt{\left(\sigma_{gi}^{2}*\sigma_{gj}^{2}\right)}$$

with *cor_ij_* the genetic correlation between mortality in populations *i* and *j*, and *cov_ij_* the genetic covariance between populations *i* and *j*.

## Results

### *F_ST_* and additive genetic relationship within and between populations

The average *F_ST_* between Savon Taimen and LUKE offspring was 0.03, indicating that on average the population difference explained 3% of the variation in allele frequencies across all markers. Based on this figure, we consider that the 2 populations were closely related.

Pairwise additive genetic relationship between the offspring of Savon Taimen population ranged between 0 and 0.70, with a median of 0.04 and a mean of 0.06. For the offspring of the LUKE population, the pairwise additive genetic relationship ranged between 0 and 0.76, with a median of 0.02 and a mean of 0.05. For the Savon Taimen fish, 62% of the pairwise comparisons were greater than 0, with a median value of 0.075. For the LUKE population, 56% of the pairwise comparisons were greater than 0, with a median value of 0.063. These figures indicate that, in both populations, the median genetic relationship between samples that are related broadly matches the value of half first cousins (0.0625).

When comparing the 2 populations, the additive genetic relationship between Savon Taimen offspring and LUKE offspring ranged between 0 and 0.34, with median of 0.00 and mean of 0.01. Comparing the additive genetic relationship between the 2 populations, we also noted that 20% of the pairwise comparisons were greater than 0, with a median value of 0.05, slightly below the value of half first cousins, based on the chain counting rule used in pedigrees (Lynch and Walsh 1997). Very importantly, every single fish in both populations has at least one nonzero additive genetic relationship value with a fish in the other population.

### MLMA results for Omy03 QTL

The MLMA analysis identified a significant (above genome-wide Bonferroni correction) peak in association of mortality with SNPs on chromosome Omy03 when all samples were pooled together (Table 1a), similarly to what was observed by Fraslin *et al.* (2022) for the LUKE samples alone. When the 2 populations and the 2 sexes are analyzed separately, we observe the same peak on chromosome Omy03 in both the LUKE female and LUKE male samples at the level of chromosome-wide significance. The Savon Taimen samples show a peak in the same location, that does not reach significance, either genome, or chromosome-wide (Table 1a).

**Table 1.** The most significant SNPs with association to mortality in genome-wide association analyses.

			Alleles		LUKE females			LUKE males			Savon Taimen			All fish together			Savon Taimen and LUKE males		
SNP	chr	bp	Major	Minor	beta ± SE	Variance explained	*P*	beta ± SE	Variance explained	*P*	beta ± SE	Variance explained	*P*	beta ± SE	Variance explained	*P*	beta ± SE	Variance explained	*P*
(a) Omy03, MLMA																			
AX-89974385	3	64,017,036	T	C	0.09 ± 0.02	0.10	1.98E-04	0.1 ± 0.02	0.13	9.94E-06	0.10 ± 0.03	0.09	1.95E-04	0.10 ± 0.02	0.10	2.56E-10	0.11 ± 0.02	0.11	1.16E-08
(b) Omy03, MLMA-LOCO																			
AX-89974385	3	640,17,036	T	C	0.09 ± 0.02	0.10	4.70E-05	0.10 ± 0.02	0.12	2.25E-06	0.11 ± 0.03	0.09	4.58E-05	0.11 ± 0.01	0.11	7.19E-14	0.11 ± 0.02	0.11	1.22E-10
AX-89953544	3	64,032,477	A	G	0.08 ± 0.02	0.08	2.07E-04	0.09 ± 0.02	0.09	5.87E-05	0.11 ± 0.04	0.06	1.22E-03	0.10 ± 0.02	0.08	1.14E-10	0.10 ± 0.02	0.08	1.50E-07
AX-89976032	3	65,733,733	G	A	0.08 ± 0.02	0.07	1.82E-03	0.08 ± 0.02	0.08	1.23E-04	0.08 ± 0.03	0.04	3.48E-03	0.08 ± 0.02	0.07	1.47E-08	0.09 ± 0.02	0.06	7.72E-07
(c) Omy05, MLMA-LOCO																			
AX-89941920	5	33,328,665	T	C	0.01 ± 0.03	0.001	6.17E-01	0.04 ± 0.02	0.03	1.78E-02	0.15 ± 0.03	0.16	1.03E-06	0.08 ± 0.02	0.04	9.25E-06	0.11 ± 0.02	0.07	5.76E-07
AX-89942562	5	50,326,041	T	G	0.02 ± 0.03	0.004	4.48E-01	0.05 ± 0.02	0.06	2.10E-03	0.10 ± 0.03	0.09	1.29E-04	0.07 ± 0.02	0.04	5.23E-06	0.09 ± 0.02	0.07	8.52E-07
AX-89957539	5	54,795,322	A	G	0.00 ± 0.03	0.000	8.85E-01	0.04 ± 0.02	0.05	2.80E-03	0.13 ± 0.03	0.14	5.69E-06	0.07 ± 0.02	0.04	1.37E-05	0.10 ± 0.02	0.08	2.67E-07
AX-89931734	5	55,555,433	A	G	0.01 ± 0.03	0.002	6.31E-01	0.05 ± 0.02	0.03	1.08E-02	0.14 ± 0.03	0.13	1.28E-05	0.07 ± 0.02	0.03	2.63E-05	0.10 ± 0.02	0.06	1.78E-06

The results are grouped by sex and population, and by 2 combined datasets (all samples together, and Savon Taimen and LUKE males together). (a) MLMA analysis, chromosome Omy03. (b) MLMA-LOCO analysis, chromosome Omy03. (c) MLMA-LOCO analysis, chromosome Omy05.

For the main SNP on Omy3 (Table 1a), the beta value for the SNP shows a positive value, indicating that the minor allele is associated with increased mortality, and in all cases the beta is on average 0.10 (range 0.09–0.11) irrespective of the *P*-value. The most significant SNP on Omy03 explains on average 10% (range 9–13%) of the additive genetic variance observed in mortality.

### MLMA-LOCO results for Omy03 QTL

**Fig. 1.**
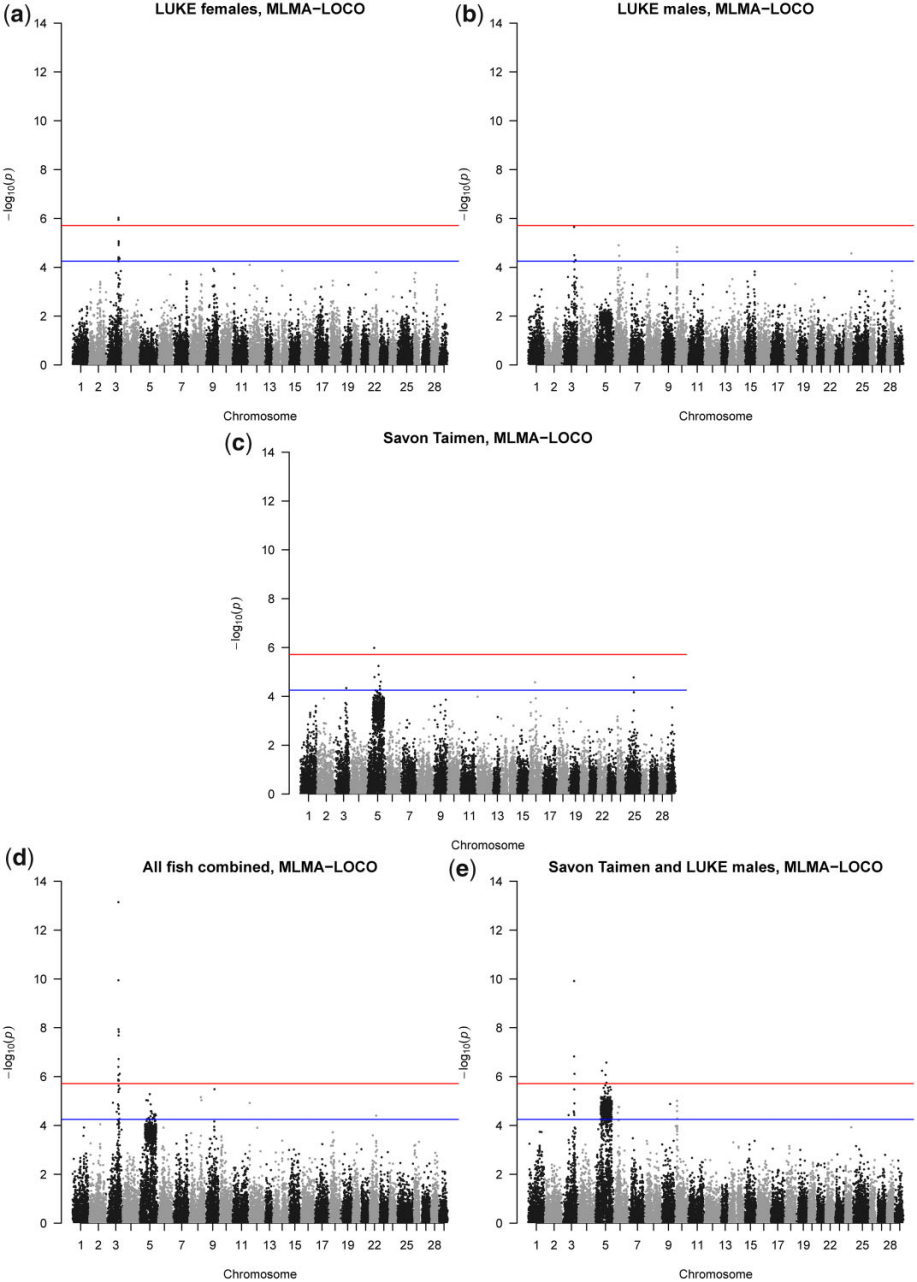
Genome-wide association analysis across all chromosomes from the MLMA-LOCO (leave-one-chromosome out) approach. For all panels, in blue the chromosome significance *P-*value, in red the genome-wide significance line, both based on Bonferroni correction. a) Manhattan plot of LUKE female fish. b) Manhattan plot of LUKE male fish. c) Manhattan plot of Savon Taimen female fish. d) Manhattan plot of all fish combined. e) Manhattan plot of Savon Taimen females and LUKE males combined.

**Fig. 2.**
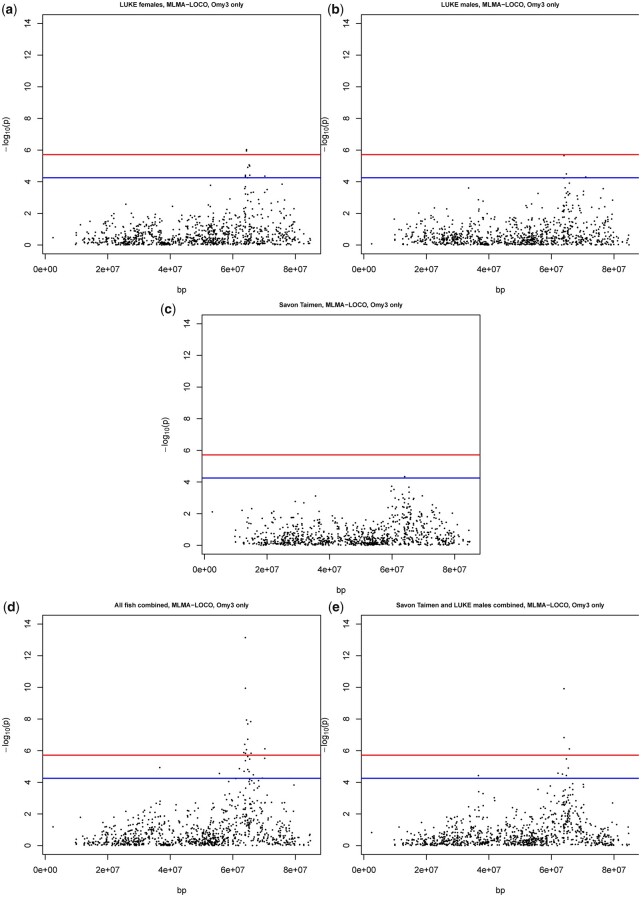
Manhattan plots for chromosome Omy3 from MLMA-LOCO analysis. For all panels, in blue the chromosome significance *P*-value, in red the genome-wide significance line, both based on Bonferroni correction. a) Manhattan plot of LUKE female fish. b) Manhattan plot of LUKE male fish. c) Manhattan plot of Savon Taimen fish. d) Manhattan plot of all fish combined. e) Manhattan plot of Savon Taimen and LUKE males combined.

We observed that LUKE females (Figs. 1a and 2a), LUKE males (Figs. 1b and 2b), and Savon Taimen fish (Figs. 1c and 2c) all show the presence of QTL on chromosome Omy03, with *P*-values reaching at least chromosome-wide level. When all samples are combined (Fig. 1, d and e) and when Savon Taimen fish and LUKE males are combined (Fig. 2, d and e), the QTL reaches genome-wide significance. Thus, this QTL is present in both sexes and populations (Table 1b).

In all analyses, the betas of the most significant SNPs indicate an average 9% increase in mortality for each rare allele substitution (beta values range 0.08–0.11) (Table 1b). The proportion of genetic variance explained by the top SNPs is 0.09–0.13.

### MLMA-LOCO identifies a large Omy05 QTL

**Fig. 3.**
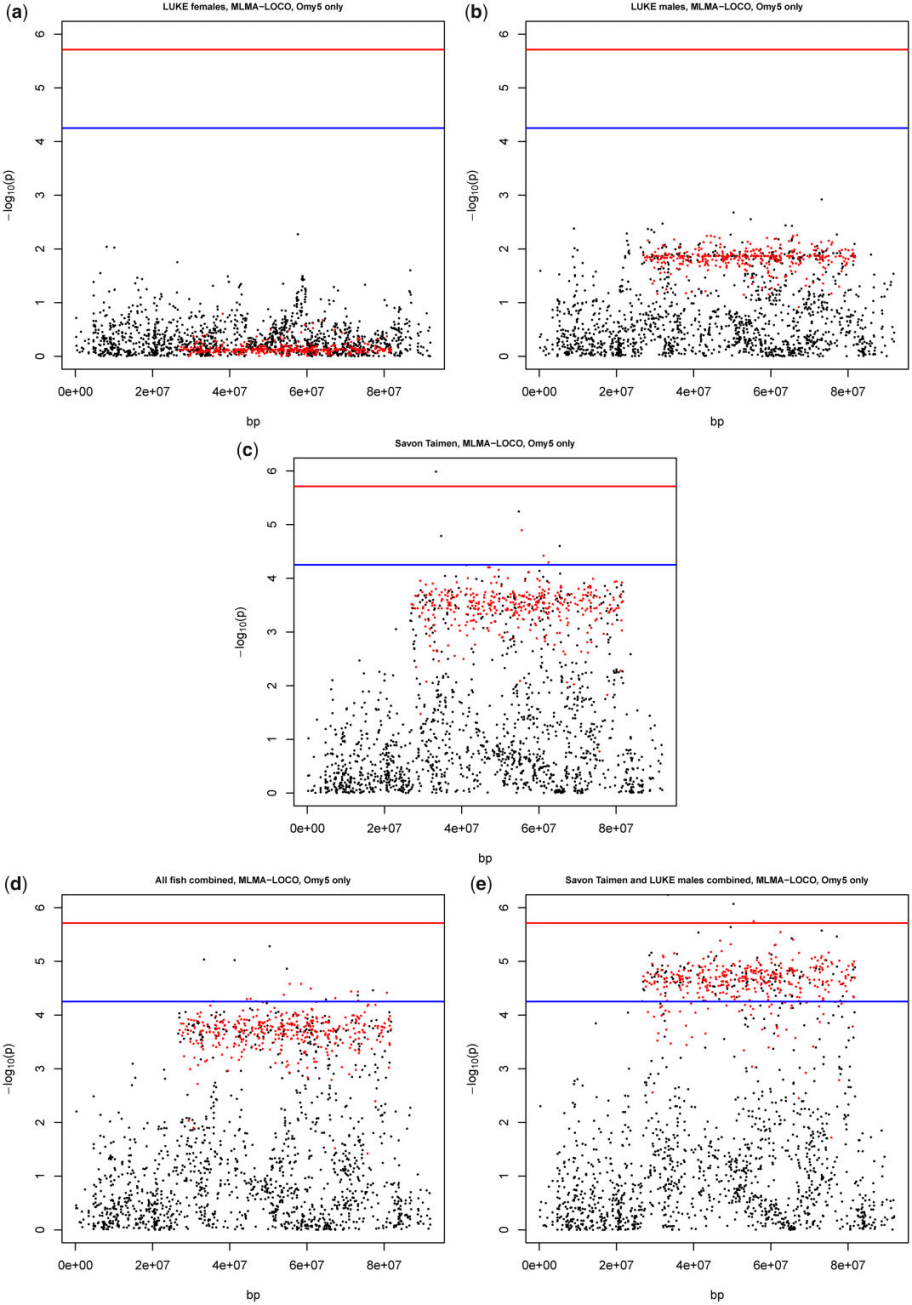
Manhattan plots for chromosome Omy5 from MLMA-LOCO analysis. For all panels, in blue the chromosome significance *P*-value, in red the genome-wide significance line, both based on Bonferroni correction. The red dots correspond to the *P*-values of SNPs marked as tagging SNPs for the large inversion complex on Omy5. a) Manhattan plot of LUKE female fish. b) Manhattan plot of LUKE male fish. c) Manhattan plot of Savon Taimen fish. d) Manhattan plot of all fish combined. e) Manhattan plot of Savon Taimen and LUKE males combined.

We observed that LUKE females (Figs. 1a and 3a) have no QTL on chromosome Omy05, LUKE males show a long flat QTL (55 Mb in length) that does not reach significance (Figs. 1b and 3b), and Savon Taimen fish show a strong long QTL containing SNPs with *P*-values reaching chromosome-wide and genome-wide significance (Figs. 1c and 3c). In the analysis combining all samples, the long QTL contains multiple SNPs with *P*-values reaching chromosome-wide significance (Figs. 1d and 3d). In the dataset combing Savon Taimen and LUKE males, the long QTL reaches chromosome-wide significance and contains multiple SNPs with *P*-values reaching genome-wide significance (Figs. 1e and 3e). The results show that the QTL is present in Savon Taimen fish, and hint that it may exist also in LUKE males. The QTL corresponds to the genetic region affected by the Omy05 inversion (in Fig. 3, the SNPs that tag the inversion are shown in red). In both populations, and in the LUKE males and LUKE females separately, the linkage between SNPs is very high (median value for all fish combined *r*^2^ = 0.75; in both LUKE males and LUKE females, and all LUKE samples the median value *r*^2^ = 0.77; in Savon Taimen median value *r*^2^ = 0.76).

In those cases where SNPs showed *P*-values above chromosome-wide or genome-wide level, the betas indicate that the effect on mortality is on average 10% (beta values range 0.07–0.15) (Table 1c), but in LUKE females close to zero (0.00–0.02). The proportion of genetic variance explained by the top SNPs is 0.09–0.16 for Savon Taimen fish, 0.03–0.06 for LUKE males, and 0.000–0.004 for LUKE females.

We identified that in our data the R haplotype has a frequency of 16%, and the A haplotype has a frequency of 84% (Table 2). When looking at the raw data, even a simple chi-square test shows an association with mortality for the R haplotype (*χ*-squared = 19.49, df = 2, *P*-value = 5.85e-05). The A haplotype shows consistent association with reduced mortality in the Savon Taimen data and in the LUKE males data (Table 2).

**Table 2.** Distribution of the genotyped samples by population, sex, and survival outcome, subdivided by the Omy05 inversion genotypes (AA for homozygote ancestral, AR for heterozygote, and RR for homozygote rearranged genotypes).

	Savon Taimen				LUKE							
	Females				Males				Females			
Omy5 genotype	A/A	A/R	R/R	Total	A/A	A/R	R/R	Total	A/A	A/R	R/R	Total
Alive (0)	635	208	7	850	501	171	8	680	486	174	23	683
Dead (1)	268	148	18	434	448	194	19	661	472	161	21	654
Mortality	0.30	0.42	0.72		0.47	0.53	0.70		0.49	0.48	0.48	

The last line provides the mortality for each population and sex, according to each Omy05 inversion genotype.

### MLMA-LOCO results for Omy06, Omy09, and Omy10 QTLs

In other chromosomes excluding Omy03 and Omy05, we do not observe any other QTL reaching genome-wide significance (Fig. 1). SNPs that are found to be chromosome-wide significant in more than one analysis are SNPs on chromosomes Omy06 and Omy10, with chromosome-wide results found in LUKE males, and in the combined Savon Taimen and LUKE males data. In LUKE males, 2 SNPs on Omy06 are at chromosome-wide significance with positive betas (0.10 and 0.13). In the combined data of Savon Taimen and LUKE males, these 2 SNPs have a positive but lower beta (range 0.07–0.09), and 1 extra SNP with negative beta (0.07). The distances between these 3 SNPs are 3 and 4 Mb, suggesting they would not be part of the same QTL, irrespective of the directions of their effects: the linkage between these SNPs was low, ranging between *r*^2^ = 0.002 and *r*^2^ = 0.17, indicating they are not part of one linkage block. The genetic variance explained ranges from 5% to 9% for the top SNPs on Omy06 and between 4% and 11% for the top SNPs on Omy10.

One SNP on chromosome Omy09 was also found to be chromosome-wide significant in all the fish combined, and the combined Savon Taimen and LUKE males, datasets. In both cases, the beta is negative (all fish combined beta = −0.08, Savon Taimen and LUKE males beta = −0.09), explaining 4% and 5% of the genetic variance in the 2 datasets.

### Heritability and genetic correlation

We observed that heritability estimates are moderate and of the same magnitude in both populations (Table 3). The linear model estimates of *h*^2^ on the liability scale are, for LUKE *h^2^* = 0.32, SD = 0.05, and for Savon Taimen *h*^2^ = 0.35, SD = 0.08. The heritability estimates from the threshold model were similar to the ones from the linear model. From threshold model, the posterior estimates of *h*^2^ are, for LUKE *h*^2^ = 0.32, SD = 0.04, and for Savon Taimen *h*^2^ = 0.36, SD = 0.06.

**Table 3.** Genetic correlation between Savon Taimen and LUKE population for mortality, and heritability (*h*^2^) of mortality, estimated with a linear model on both observed and liability scales, and the posterior estimates obtained with a threshold model.

	Sample mean	SD
Linear model		
Genetic correlation	0.62	0.25
Savon Taimen: *h*^2^ observed scale	0.21	0.05
Savon Taimen: *h*^2^ liability scale	0.35	0.08
LUKE: *h*^2^ observed scale	0.20	0.03
LUKE: *h*^2^ liability scale	0.32	0.05
	**Posterior mean**	**Posterior sd**
Posterior estimate of threshold model		
Genetic correlation	0.64	0.23
Savon Taimen: *h*^2^	0.36	0.06
LUKE: *h*^2^	0.32	0.04

Genetic correlation between mortality in the 2 populations is positive and moderate. The linear model estimates a genetic correlation of 0.62 (SD = 0.25), and the threshold model estimates a genetic correlation of 0.64 (SD = 0.23) (Table 3).

## Discussion

Our results provide an assessment of the conservation of the genetic architecture, and the role of an extensive chromosomal inversion, underlying a disease resistance phenotype in 2 populations whose kinship has been estimated at the genome-wide level.

### Population differentiation

Selective breeding for resistance would strongly benefit from knowing whether the same loci are associated with survival in all (or most) breeding stocks, and what is the range of possible responses that can be attributed to a QTL. The quantification of the genetic distance between populations allows to create testable expectations on the permanence and effect of QTLs based on the genetic distance between stocks. To give a frame of reference to the *F_ST_* figures we obtained, we directly compared them to the *F_ST_* results from the analysis of 7 French broodstocks genotyped using the very same SNP chip we used (D’Ambrosio *et al.* 2019). Based on the *F_ST_* comparisons reported by D’Ambrosio, the *F_ST_* value we observe is of the same order of magnitude with the *F_ST_* observed between closely related French INRA broodstock, where the populations are known to be separated by about 5–10 generations. This result is also consistent with the observation that every fish in 1 population has at least 1 nonzero additive genetic relationship with the other population, with a value compatible with the generational distance we assess by *F_ST_* analysis.

Our assessment of the genetic distance between 2 stocks used in the present QTL mapping analysis informs our understanding of the degree to which the stocks share common genetic determination.

### Effect of the inversion on Omy05 on mortality in the 2 populations

The most novel result we observed is the presence of a QTL associated with mortality on the large double inversion present on chromosome Omy05. The presence of an inversion on Omy05 has been documented in the wild (Pearse *et al.* 2019) and in commercial broodstocks of rainbow trout (Gao *et al.* 2021). Our analysis revealed the presence of at least 1 QTL on the area on Omy05 affected by the inversion. This inversion is composed by 2 adjacent inversions, covering approximately 60% of the chromosome, of 21.99 and 32.83 Mb in length, respectively, the first inversion of which is pericentric. Despite the presence of 2 inversions, in practice in our data they behave as one single, large inversion, and the same behavior is also reported by Pearse *et al.* (2019).

The inversion is naturally occurring in wild populations in North America (Pearse *et al.* 2019), and it is estimated that it has been maintained in wild populations for about 1.5 million years. Different analytical approaches also suggest that the inversion is also in farmed populations in the United States (Vallejo *et al.* 2018; Gao *et al.* 2021) and France (D’Ambrosio *et al*. 2019). Both haplotypes reported in the literature, A (ancestral) and R (rearranged) are found in our data, matching the tagging SNPs reported by Gao *et al.* (2021), and we could assign every sample to the 3 possible AA/AR/RR genotypes.

In our data, the most common haplotype is the A haplotype, and it is associated with a reduced mortality to columnaris disease especially in the Savon Taimen population. Pearse *et al.* (2019) highlight how this inversion acts a “super gene” which contains multiple loci. The inversion contains a large number of genes analogous to genes whose effect has been characterized in other model organisms, and thus have a putative function in *O. mykiss*, including genes associated with circadian rhythm, photosensory, adiposity, and age at maturity. Inversions may play an important role in evolution by reducing recombination between favorable combinations of alleles. In natural populations, the A haplotype is associated with the steelhead anadromous morph in females, and R haplotype is associated with a sedentary freshwater morph in males. The A haplotype is also associated with slightly slower growth. The A haplotype, that in our data increases resistance against warm water columnaris disease, exhibits a clinal distribution in wild populations of *O. mykiss* ranging from Southern Alaska to Southern California, with the highest prevalence in southern rivers (Pearse *et al.* 2019). Capture-recapture data of wild *O. mykiss* in the Big Creek river in California show that the frequency of individuals carrying the A haplotype increases with warming water temperature (Pearse *et al.* 2019). The inversion was tagged in a wild population by 2 SNP markers by Pearse *et al.* (2019), which were further expanded in a farmed population to 475 SNPs by Gao *et al.* (2021) in a farmed stock, providing clear evidence of the conservation of this chromosomal rearrangement between wild *O. mykiss* and aquaculture stock. In our populations, these tagging SNPs match the known location and the size of the inversion, and showed association with mortality, indicating the presence of at least 1 QTL in the inversion, the exact location which will have to be further elucidated.

Our analysis of the Omy05 inversion QTL showed, however, that the magnitude of the allele substitution effect depended on sex and population. Without dividing the data in its constituent populations, and without dividing the LUKE population in males and females, the impact of Omy5 QTL would have been substantially less clear. We detected a clear effect on the Savon Taimen females (9–16% of the genetic variance explained, beta range 0.10–0.15), and nonexisting effect in the LUKE females (0.01–0.2% of the genetic variance explained, beta range 0.00–0.02). The LUKE males showed a nonsignificant effect, but the direction of the effect was similar to Savon Taimen fish (3–6% of the genetic variance explained, beta range 0.04–0.05). Moreover, by combining the data of Savon Taimen and LUKE males, the *P*-values on a −log_10_ scale of the SNPs on Omy05 were strongly elevated, implying there may be the same underlying QTL in both groups (Figs. 1 and 3 and Table 2). However, there is no immediate explanation of the discrepancy we observe between sexes and populations. There is evidence that the Omy05 inversion contains loci that are associated with sex determination and sex-specific expression of traits (Pearse *et al.* 2019), and we know that the sires of the Savon Taimen samples are sex reversed females. However, the cause of the discrepancy of effects in the 2 populations and sexes still remains unexplained.

### The QTL on Omy03 is conserved in the 2 populations

Our analysis successfully identified a QTL on chromosome 3 associated with mortality. The broad picture we can take from this analysis is that the QTL on chromosome Omy03 is strongly conserved between these 2 populations, both in terms of location, and the proportion of genetic variance explained (LUKE females 7–10%; LUKE males 8–12%; Savon Taimen 4–9%; all fish together 7–11%; LUKE males and Savon Taimen 6–11%), and with what seems to be a negligible, or limited, effect of sex on the QTL.

With the full data analyzed together, the QTL on chromosome Omy03 shows the highest *P*-value peak (on a −log_10_ scale) compared to any other locus (Figs. 1 and 2). The significance of the *P*-value for association with mortality diminished depending on what subset of the data was included in analysis, though in all cases the QTL on Omy03 reached chromosome-wide or genome-wide significance. The loss of power in resolving the QTL is obviously not at all surprising, given that fewer samples lead to less statistical power. In the LUKE samples, both males and females showed a *P*-value for association between the QTL and mortality that was broadly of the same order of magnitude, though it is less significant than when all samples are considered together. The Savon Taimen data showed a slightly lower significance compared to either sex in the LUKE data. These consistent patterns differed from the results of the Omy05.

The only previous effort to map QTL for mortality to columnaris disease by GWAS does not report association loci matching our results (Silva, Evenhuis, Vallejo, Gao, *et al.* 2019). Silva, Evenhuis, Vallejo, Gao, *et al.* (2019) used 2 breeding lines of North American rainbow trout exposed to a virulent strain of *F. columnare*, analyzed independently with the same SNP chip used here, but with an exposure/challenge experimental setup, rather than a naturally occurring outbreaks (as it is for our data). The results of that experiment identified QTLs that were mostly not overlapping in the 2 strains, and none of them were either on chromosomes Omy03 or Omy05. On the other hand, a positive correlation has been reported between resistance to columnaris disease and BCWD (Evenhuis *et al.* 2015; Silva, Evenhuis, Vallejo, Tsuruta, *et al.* 2019). Three QTLs recently reported for BCWD resistance (Vallejo, Liu, *et al.* 2017; Liu *et al.* 2018; Fraslin *et al.* 2018; Fraslin *et al.* 2019) have been identified on chromosome Omy03, a result that is comparable with our results. Nevertheless, the genome-wide significant SNPs we identified on Omy03 span from 63,527,178 to 70,261,448 bp, placing this QTL between the 2 of the reported for BCWD (the first spanning from 61,621,949 to 62,558,467 bp, the second spanning from 77,108,538 to78,076,592 bp). Our results raise the question of the degree to which the correlation between CD and BCWD is due to the fact that the same QTLs control both traits, or whether different QTLs are located in the same genomic region in linkage disequilibrium.

### Heritability and genetic correlation

We estimated heritability (on both observed and liability scales), and genetic correlation using the realized GRM generated from the genome-wide SNP data. This approach captures the full genetic contribution to the mortality to columnaris disease, rather than just the effects of the main QTLs, and is parallel to the approach that can be used in genomic evaluation to estimate genomic breeding values. The liability-scale heritability values indicate a moderate genetic effect on the phenotype, which broadly matches those previously reported for columnaris disease (0.23 and 0.34, Vallejo, Liu, *et al.* 2019), using the same SNP chip.

An advantage of our approach of using a genome-wide SNP dataset in the 2 populations is the ability to have a measure of the realized genomic relationship between individuals of both populations. This allows to directly assess the genetic correlation between populations for mortality, even for distinct populations and when the traditional sire-dam-offspring pedigree does not exist (Table 3). In our data, the genetic correlation was positive (*r_G_* = 0.62−0.64) indicating that the genetic basis of resistance is indeed partly similar in the 2 populations. Yet, these figures are likely an underestimate of the true genomic correlation between populations, given that the causal variants were likely not directly genotyped, there is recombination between SNPs and the causal variant, and the allele frequencies at the causal variant differ in the 2 populations (Wientjes *et al.* 2018). To summarize, tn line with our QTL analysis these results together reflect a moderate degree of consistency of QTL effects and of other minor polygenetic genetic effects in determining the phenotype.

### QTL validation

The conservation of QTL locations is typically ascertained by testing for the same phenotype in multiple populations (Vallejo *et al.* 2014; Evenhuis *et al.* 2015; Vallejo, Liu, *et al.* 2017; Silva, Evenhuis, Vallejo, Gao, *et al.* 2019) or generations (Liu *et al.* 2018) for the presence and location of the QTLs, as we present here with the Savon Taimen and LUKE populations. Our results indicate that mortality to columnaris disease is either oligogenic or polygenic in nature, a fact that makes QTL validation more challenging compared to monogenic traits. Nevertheless, our data does provide evidence that some QTLs associated with decreased mortality to columnaris disease are conserved in the 2 separate populations of rainbow trout, supporting the hypothesis that this trait is indeed controlled by few replicable QTLs (that explain around 10% of genetic variation) and the remaining polygenetic effects. There are 2 mutually nonexclusive reason for finding similar QTLs in the 2 populations. First, the most significant SNPs may be strongly linked to the causal variant, with minimal chances of recombination breaking the linkage between SNPs and causal variant. Second, the close kinship between the 2 populations in our analysis suggests that the genetic architecture for a phenotype will overlap.

The large inversion on Omy05 provides one special case of a genomic region with limited or no recombination. The effect of this inversion is to create a long haplotype with very strong or complete linkage disequilibrium between all markers. Any QTL located on this inversion can be effectively tagged by any and all the SNPs known to tag the inversion, making allele detection for the inversion extremely easy and reliable.

### Marker-assisted selection and genomic selection

Our results show that there is potential to improve resistance against *F. columnare* by both genomic selection and MAS. To date, the most successful implementations of MAS in aquaculture are the well-publicized infectious pancreatic necrosis (*IPN*) case (Houston *et al.* 2010; Moen *et al.* 2009), resistance for lymphocystis disease in Japanese flounder (Fuji *et al.* 2007), and, potentially, rainbow trout selected for resistance to BCWD using 2 QTLs (these are commercially marketed by Aquagen, but no results have been published in the literature). The *VGLL3* locus (Ayllon *et al.* 2015; Barson *et al.* 2015) could also be used for selection of maturity and growth traits in European stocks of Atlantic salmon (Debes *et al.* 2021), but most likely not in North American ones, because of the different genetic architecture of traits in North American stocks of Atlantic salmon (Boulding *et al.* 2019). In our data, we observe that the main QTL explains 7–11% of the genetic variance with the extreme genotypes having a 8–11% difference in mortality, implying they would be also a valid choice for MAS for decreased mortality. The inversion on Omy05 would be an effective target of MAS, because it can be extremely reliably tagged, and because tagging markers are unlikely to decrease their tagging potential even over a long time period because the inversion precludes recombination. The presence of QTLs for decreased mortality, and the opportunity to use MAS in a warming climate, where outbreaks of columnaris disease are more likely, can have substantial practical implications.

GS that uses genome-wide marker sets is a second viable approach, and it allows to estimate a genomic breeding value for each sample, the GEBV (Meuwissen *et al.* 2001; Goddard and Hayes 2007). GS is especially beneficial for polygenic traits, because the effect of each locus is small, and thus MAS would not be very effective (Meuwissen *et al.* 2001). GS provides improvement over pedigree-based evaluations, for instance in the LUKE population, GS shows an 13.6% increase in the accuracy of GEBVs for resistance to *F.* *columnare* infection compared to the use of pedigree data alone (Fraslin *et al.* 2022). By specifically modeling the effects of SNPs on phenotype in GS, selection accuracies can be potentially increased further when QTLs exists (Fraslin *et al.* 2022). Effectiveness of GS depends on the presence of a dense set of genome-wide markers capturing all haplotype blocks across the genome and thus all possible QTLs affecting the trait (Meuwissen *et al.* 2001; Goddard and Hayes 2007), and on a sufficiently large reference population (VanRaden *et al.* 2009). Yet, because of the potential costs of genotyping many thousands of markers in thousands of samples, current attempts of using a much smaller subsets of SNPs than those present in a SNP chip have the potential of major savings. It is theoretically possible to impute a genome-wide marker set based on the genotyped markers, obtaining the benefits of a whole genome scan at a lower cost (Dufflocq *et al.* 2019; Tsairidou *et al.* 2020). It is also possible to use these marker sets to reconstruct pedigrees, for a single step GBLUP approach. Nevertheless, a much smaller subset of SNPs might prove problematic in reconstructing the GRM (Misztal *et al.* 2020) and might not capture all loci involved in the phenotype, and thus it might decrease the effectiveness of selection approaches. Despite these concerns, empirical evidence shows that small marker sets with markers chosen to tag known QTLs can provide better power and more accurate genomic predictions than pedigree based approaches, at least for disease resistance (Vallejo, Leeds, *et al.* 2017; Vallejo et al. 2018). Aquaculture species have been only recently domesticated and subject to selection, which might also explain the presence of QTLs explaining a large amount of variation for many commercially relevant traits (Houston *et al.* 2020), due to the fact selection for the most important QTLs has not brought them to fixation.

To conclude, our results bring an important insight into this discussion. We show that resistance to columnaris disease is oligogenic in the 2 populations we analyzed, with QTL conservation between them. Additionally, our results show the potential importance of genomic rearrangements in the genetic architecture of a trait. Genomic rearrangements reduce or ablate recombination and can be successfully tagged by one or 2 SNPs, with fewer chances that recombination would weaken the linkage between QTL and tagging SNPs over generations. Our results also show that, while inversions can play an important role in determining a phenotype and can have potential to be used to augment selective breeding, their actual effect needs to be characterized on a case by case.

## Ethic statement

The establishment of progeny families at LUKE’s facilities followed the protocols approved by the LUKE’s Animal Care Committee, Helsinki, Finland. Savon Taimen Oy and Hanka-Taimen Oy, 2 fish farming companies, have authorization for fish management, rearing and experiments, and all parties comply with the EU Directive 2010/63/EU for animal experiments.

## Data availability

Data are made available on figshare at the address: https://doi.org/10.6084/m9.figshare.19323506.

## Conflicts of interest

None declared.
